# *Ginkgo biloba* Leaf Extract Protects against Myocardial Injury via Attenuation of Endoplasmic Reticulum Stress in Streptozotocin-Induced Diabetic ApoE^−/−^ Mice

**DOI:** 10.1155/2018/2370617

**Published:** 2018-02-25

**Authors:** Jinfan Tian, Yanfei Liu, Yue Liu, Keji Chen, Shuzheng Lyu

**Affiliations:** ^1^Department of Cardiology, Beijing Anzhen Hospital, Capital Medical University, Beijing 100029, China; ^2^Beijing Institute of Heart, Lung and Blood Vessel Diseases, Beijing 100029, China; ^3^Cardiovascular Disease Center, Xiyuan Hospital, China Academy of Chinese Medical Sciences, Beijing 100091, China; ^4^Graduate School, Beijing University of Chinese Medicine, Beijing 100029, China

## Abstract

Diabetes was induced in high-fat diet-fed ApoE^−/−^ mice via administration of low-dose streptozotocin (STZ) for five days. Mice were then treated with GBE (200 or 400 mg/kg) by gastric gavage daily for 12 weeks. Mice in the untreated diabetic group received saline instead, and nondiabetic C57BL/6J mice served as controls. Collagen І and ІІІ mRNA expression was measured by real-time PCR. TNF-*α*, IL-1*β* mRNA levels, and NF-*κ*B expression were determined to analyze intramyocardial inflammation. Hallmarks of endoplasmic reticulum stress- (ERS-) related apoptosis pathways, including phosphorylated c-Jun N-terminal kinase (p-JNK), C/EBP homologous protein (CHOP), caspase-12, and cleaved caspase-3, were analyzed by Western blotting. Diabetic ApoE^−/−^ myocardial injury was associated with increased cardiomyocyte apoptosis (increased expression of p-JNK, CHOP, caspase-12, and cleaved caspase-3), interstitial fibrosis (increased mRNA levels of collagen І and ІІІ), and inflammation (increased mRNA levels of TNF-*α* and IL-1*β*, and NF-*κ*B expression). GBE at 200 and 400 mg/kg/day significantly attenuated cardiomyocyte apoptosis, collagen deposition, and inflammation in diabetic mice via inhibition of the p-JNK, CHOP, and caspase-12 pathways. Serum levels of the proinflammatory cytokines (IL-6, IL-1*β*, and TNF-*α*), blood glucose, and lipid profiles were also regulated by GBE treatment. GBE might be beneficial in the treatment of diabetic myocardial injury.

## 1. Introduction

Diabetic cardiomyopathy (DCM), one of the leading cardiovascular complications of diabetes, ultimately leads to heart failure, which increases the mortality among diabetes patients. Diabetic myocardial injuries, including cardiomyocyte apoptosis, myocardial fibrosis, and intramyocardial inflammation, are important pathological characteristics of DCM. Diabetes-induced cardiomyocyte apoptosis often occurs concomitantly with interstitial collagen deposition and myofiber disarray [1]. In addition, accumulating evidence has shown that substrate metabolic alteration, oxidative stress, and chronic inflammation contribute to DCM and diabetic myocardial injury [2, 3].

Endoplasmic reticulum stress (ERS) plays a critical role in the development of diabetic myocardial injury because the sustained and uncorrected unfolded protein response (UPR) could induce cell death [4]. The UPR is mediated by three pathways, the inositol-requiring kinase-1 (IRE1), protein kinase R-like ER kinase (PERK), and activating transcription factor 6 (ATF6) pathways. ERS-mediated cell death involves activation of c-Jun *N*-terminal kinase (JNK), C/EBP homologous protein (CHOP), and caspase-12, which consequently activates caspase-3.

IRE1 and JNK activation may result in the upregulation of nuclear factor kappa-B (NF-*κ*B) expression via phosphorylation of IkB kinase (IKK) [5, 6]. Upregulation of NF-*κ*B expression leads to increased production of proinflammatory cytokines, such as tumor necrosis factor-*α* (TNF-*α*), interleukin-1*β* (IL-1*β*), and IL-6, which contribute to cardiomyocyte apoptosis and myocardial fibrosis [7].

**Figure 1:**
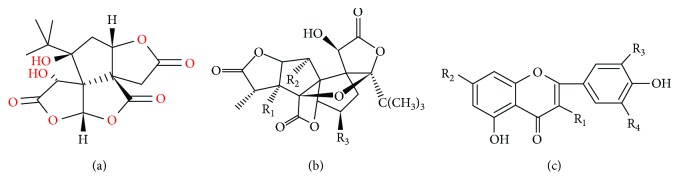
Chemical structures of the constituents of GBE. (a) Bilobalide, (b) ginkgolide, and (c) ginkgo flavonol glycosides.

Recently, herbal treatment of diabetic myocardial injury has gained much attention. *Ginkgo biloba* leaves have been used as a traditional herbal medicine for hundreds of years in China. The major components of *Ginkgo biloba* leaf extract (GBE) include two active substances, namely, terpenoids (including ginkgolides and bilobalide) and flavonoids (Figure 1). GBE was shown to exhibit antioxidant, free radical scavenging and membrane-stabilizing activities, which contributed to its beneficial effects in ischemia/reperfusion injury in a diabetic rat myocardium [8]. In addition, it showed anti-inflammatory and antioxidant effects in the pancreas of streptozotocin- (STZ-) induced diabetic animals [9, 10]. Moreover, GBE enhanced insulin sensitivity and prevented insulin resistance by increasing insulin-induced Akt phosphorylation and insulin receptor substrate 1 expression [11]. GBE was also shown to ameliorate diabetic nephropathy in STZ-induced diabetic rats [12].

Currently, few studies have investigated the potential use of GBE for the treatment of diabetic myocardial injury. In the present study, we aimed to investigate whether GBE could protect against diabetic myocardial injury and elucidate the underlying mechanisms.

## 2. Materials and Methods

### 2.1. Drugs

GBE powders and atorvastatin were purchased from Beijing Handian Pharmaceutical Co. Ltd. and Pfizer Pharmaceutical Co. Ltd., respectively. GBE used in the present study contains 44.9% ginkgo flavonoids, 6.3% terpenoids, and <1 ppm ginkgo acid.

### 2.2. Experimental Animals

Male ApoE^−/−^ mice, aged 6-7 weeks and weighing 19–21 g (C57BL/6J background, introduced from Jackson Laboratory of USA by Peking University Health Science Center Laboratory Animal Science Department; quality certification number SCXK (Beijing) 2016-0012), were used in this study. The rearing condition of the mice was grade 2. Mice were maintained under controlled conditions (room temperature, 22–24°C; relative humidity, 50%; and lights on; from 7:00 to 19:00). The experimental protocol was approved by the institutional animal care and use committee of Xiyuan Hospital, China Academy of Chinese Medical Sciences. Animal experiments were carried out in accordance with the Guide for the Care and Use of Laboratory Animals published by the US National Institutes of Health.

### 2.3. Experimental Protocol

**Figure 2:**
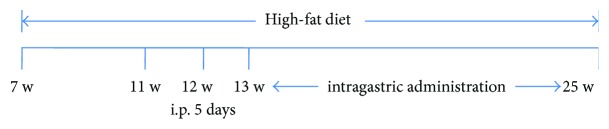
Timeline of the experimental protocol *in vivo*.

ApoE^−/−^ mice were fed with a high-fat diet (basic diet, 78.85%; fat, 21%; and cholesterol, 0.15%) for four weeks before diabetes was induced by intraperitoneal injection of 50 mg/kg/day STZ (Sigma) diluted with citrate buffer (pH 4.5; final concentration, 1%) for five consecutive days, as described in a previous study [13]. Mice exhibiting plasma glucose levels > 12 mmol/L were considered diabetic and were used in the study (*n* = 58) [13, 14]. The diabetic mice were then treated with atorvastatin [15, 16] (10 mg/kg/day, intragastric (i.g.), *n* = 14), GBE at a low dose (200 mg/kg/day, i.g., *n* = 16), or GBE at a high dose (400 mg/kg/day, i.g., *n* = 17). The doses of GBE were selected based on previous studies [17, 18]. Diabetic mice treated with equal volumes of saline served as the untreated diabetic group (*n* = 11). All ApoE^−/−^ mice were maintained on a high-fat diet and sacrificed after 12-week treatment. C58BL/6J mice (*n* = 20) served as the control group. The study timeline is shown in Figure 2.

### 2.4. Body Weight and Plasma Glucose Changes

The body weight and fasting plasma glucose levels were measured before the initial GBE dose and every four weeks thereafter. Plasma samples were collected by the cutting tail method, and the plasma glucose levels were measured using a glucometer (Roche).

### 2.5. Tissue Preparation and Histological Examination

All animals were euthanized, and the heart samples were collected before they were perfused with heparin saline. The specimens were transversely cut and fixed with 4% paraformaldehyde for 24 h. They were then embedded in paraffin and cut into 5 *μ*m thick sections for hematoxylin/eosin and Masson's staining. Immunohistochemical staining of cleaved caspase-3 was also performed (rabbit polyclonal anti-cleaved caspase-3, CST, 1 : 200 dilution). Immunohistochemical semiquantitative analysis was conducted on microscopic images using Image-pro plus 6.0 software (Media Cybernetics Inc., Rockville, MD, USA) under 200x magnification. The positive expression of cleaved caspase-3 was represented by integral optical density (IOD).

### 2.6. Western Blot Analysis

The heart tissues were removed from liquid nitrogen, weighed, and homogenized in radioimmunoprecipitation assay (RIPA) lysis buffer. Protein concentration was determined using the bicinchoninic acid method. Equal amounts of protein (40 *μ*g) from each sample were separated by sodium dodecyl sulfate-polyacrylamide gel electrophoresis (SDS-PAGE) and transferred onto a nitrocellulose membrane. Nonspecific sites were blocked by incubating the membranes with 5% nonfat milk and 0.2% tween 20 in Tris-buffered saline for 2 h at room temperature. After washing, the membranes were incubated overnight at 4°C with the following primary antibodies: anti-CHOP (CST2895S, 1 : 2000), anti-JNK (CST9252S, 1 : 2000), anti-p-JNK (CST9251S, 1 : 1000), anti-caspase-12 (CST2202S, 1 : 1000), anti-cleaved caspase-3 (CST9664S, 1 : 1000), and anti-NF-*κ*B (Abcam 86299, 1 : 2000). The membranes were washed with TBS-T and incubated with horseradish peroxidase- (HRP-) conjugated secondary antibodies. Then, the membrane was assayed using an enhanced chemiluminescence system. Glyceraldehyde 3-phosphate dehydrogenase (GAPDH) was used to ensure equal sample loading. The expression levels of CHOP, caspase-12, cleaved caspase-3, and NF-*κ*B were adjusted for GAPDH, and the values were normalized over the untreated diabetic group. The expression levels of p-JNK were adjusted for total JNK and then normalized over the untreated diabetic group.

### 2.7. Quantitative Real-Time PCR

Real-time polymerase chain reaction (PCR) was performed to determine the mRNA expression of collagen I and III, TNF-*α*, and IL-1*β*. GAPDH was used as an internal control. The primer sequences were as follows: collagen I, 5′-TGGAAACCCGAGGTATGCTT-3′(forward) and 5′-CATTGCATTGCACGTCATCG-3′ (reverse); collagen III, 5′-ACTGGTGAACGTGGCTCTAA-3′ (forward) and 5′-AACCTGGAGGACCTGGATTG-3′ (reverse); TNF-*α*, 5′-CTCATGCACCACCATCAAGG-3′ (forward) and 5′-ACCTGACCACTCTCCCTTTG-3′ (reverse); IL-1*β*, 5′-GAAGAAGAGCCCATCCTCTG-3′ (forward) and 5′-TCATCTCGGAGCCTGTAGTG-3′ (reverse); and GAPDH, 5′-TGCCCCCATGTTTGTGATG-3′ (forward) and 5′-TGTGGTCATGAGCCCTTCC-3′(reverse). Relative mRNA level was normalized over the untreated diabetic group. All experiments were repeated for at least three times.

### 2.8. Serum Lipid Profile and Glucose Analysis

At the end of the 12-week period, all mice were fasted overnight before they were sacrificed, and blood samples were collected and centrifuged at 3000 rpm for 10 min. Serum glucose, high-density lipoprotein cholesterol (HDL-c), total cholesterol (TC), triglycerides (TG), and low-density lipoprotein cholesterol (LDL-c) levels were determined using an automated system.

### 2.9. Measurement of Serum Inflammatory Cytokine Levels

Serum levels of inflammatory cytokines (IL-6, IL-1*β*, and TNF-*α*) were measured using commercially available ELISA kits, purchased from Beijing Fang Cheng Jia Hong Technology Co. Ltd. (catalog numbers FU-X0850, FU-X0840, and FU-X1059, resp.). The serum was collected as previously described. Five serial dilutions of the standard were prepared according to the manufacturer's instructions. Blank and sample wells were set, respectively. Sample diluent (40 *μ*L) was added to the sample wells in the precoated ELISA plates, followed by the addition of the samples (10 *μ*L). After sealing the plates with a closure plate membrane, they were incubated for 30 min at 37°C. HRP-conjugated reagent (50 *μ*L) was added to all wells, except for the blank well. After incubation at 37°C, the liquid in the wells was removed, and the plate was washed with a wash liquid. Chromogen solution A (50 *μ*L) and chromogen solution B (50 *μ*L) were added to each well. The plates were incubated in dark at 37°C for 15 min. The blank well was considered zero, and the absorbance of each well was measured at 450 nm within 15 min after adding the stop solution.

### 2.10. Statistical Analysis

SPSS 17.0 was used for statistical analyses. The data were presented as the means ± standard deviation ($\bar{x}\pm s$). One-way analysis of variance (ANOVA) was used to perform comparisons among group means, and the least significant difference (LSD) test was used for multiple comparisons between the untreated diabetic group and other groups. *P* < 0.05 was considered statistically significant. GraphPad Prism 5.0 software was used for graphical presentation.

## 3. Results

### 3.1. Body Weight and Plasma Glucose Levels

STZ resulted in a significant increase in plasma glucose levels, compared to those in the control group (14.8 ± 2.2 versus 5.3 ± 0.8 mmol/L, *P* < 0.01). At the end of the 12-week gavage, the untreated diabetic mice showed severe hyperglycemia compared to the control group (23.4 ± 6.4 versus 7.5 ± 1.0 mmol/L, *P* < 0.01). GBE treatment at 200 and 400 mg/kg/day suppressed the plasma glucose levels; however, only high-dose GBE resulted in a statistically significant difference (low-dose GBE group versus untreated diabetic group, 18.8 ± 6.5 mmol/L versus 23.4 ± 6.4 mmol/L, *P* = 0.06; high-dose GBE group versus untreated diabetic group, 15.3 ± 7.1 mmol/L versus 23.4 ± 6.4 mmol/L, *P* = 0.01).

There was a significant weight loss in the diabetic mice compared to those in the control group (22.37 ± 11.67 versus 26.58 ± 11.56 g, *P* < 0.01). Body weight loss was associated with hyperglycemia and polyuria. Body weight of mice in the untreated diabetic group was significantly lower than that in the control group at the end of the study course (30.01 ± 1.35 versus 26.38 ± 22.72 g, *P* < 0.01). Atorvastatin and GBE treatment did not affect the body weight in diabetic mice.

### 3.2. Effect of GBE on Serum Lipid and Glucose Profiles

**Figure 3:**
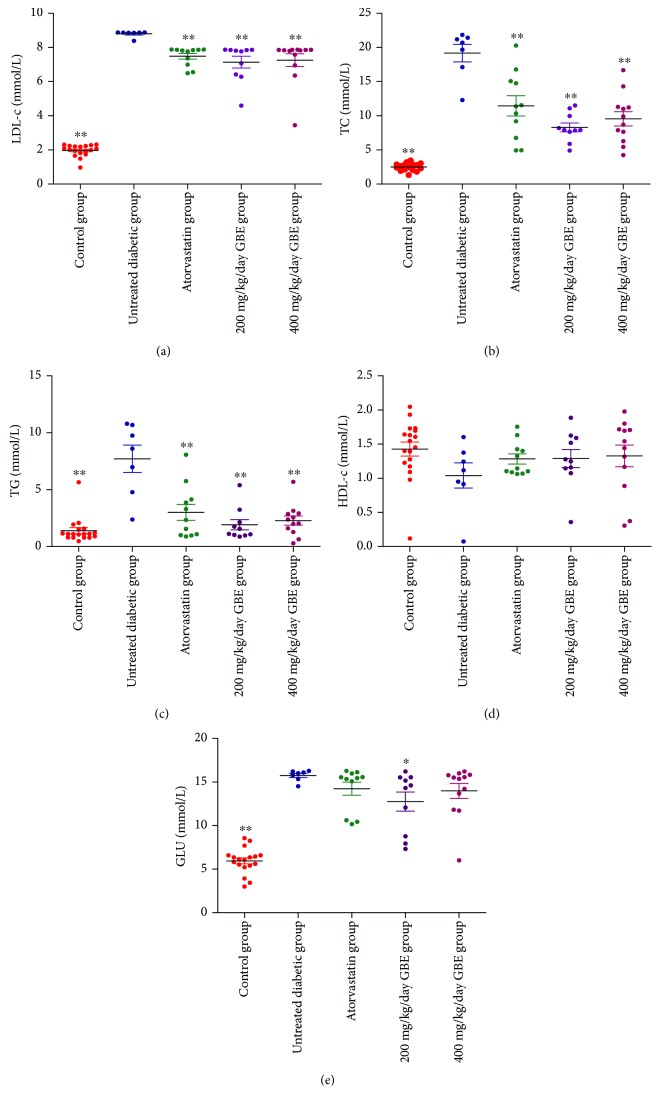
Serum lipid profiles and glucose levels. ^∗^*P* < 0.05 and ^∗∗^*P* < 0.01 versus the untreated diabetic group.

Serum lipid and blood glucose levels were measured before the mice were sacrificed. LDL-c, TC, TG, and blood glucose levels significantly increased in the untreated diabetic group, compared to those in the control group. Atorvastatin and GBE (200 and 400 mg/kg/day) significantly decreased LDL-c, TC, and TG levels (*P* < 0.01, Figures 3(a)–3(c)). GBE at 200 mg/kg/day lowered the serum glucose levels, compared to those in the untreated diabetic group (*P* < 0.05, Figure 3(e)). There were no significant differences in HDL-c levels among the control, untreated diabetic, atorvastatin, low-dose GBE, and high-dose GBE groups (Figure 3(d)).

### 3.3. Effect of GBE on Serum Inflammatory Cytokine Levels

**Figure 4:**
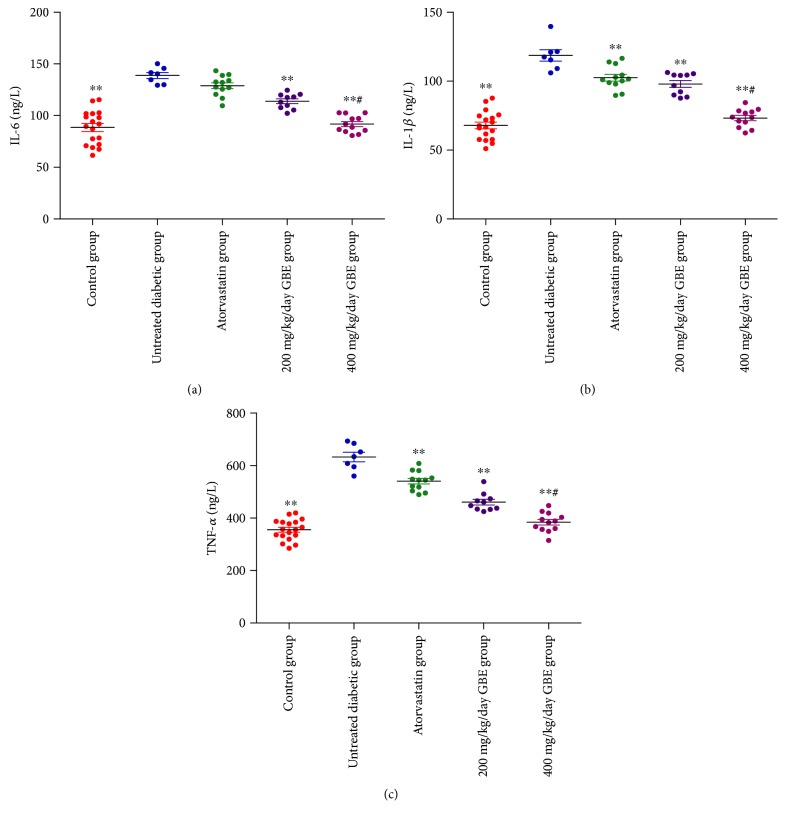
Serum proinflammatory cytokines determined by ELISA. ^∗∗^*P* < 0.01 versus the untreated diabetic group; ^#^*P* < 0.01 versus the 200 mg/kg/day GBE group.

The levels of serum inflammatory cytokines, including IL-6, IL-1*β*, and TNF-*α*, significantly increased in the diabetic mice, compared to the control mice. GBE (200 and 400 mg/kg/day) significantly decreased serum IL-1*β*, TNF-*α*, and IL-6 levels. Moreover, high-dose GBE (400 mg/kg/day) resulted in lower levels of inflammatory cytokines, compared to those administered with low-dose GBE (*P* < 0.01, Figures 4(a)–4(c)).

### 3.4. Effect of GBE on the Histomorphology of Diabetic Hearts

**Figure 5:**
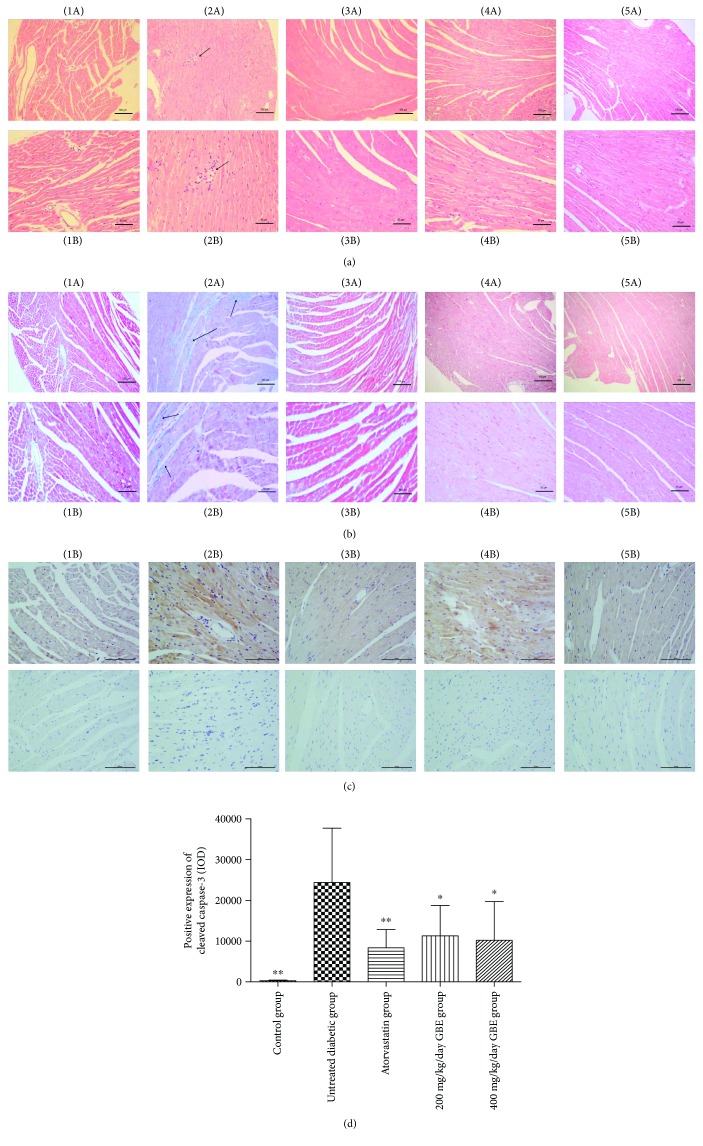
Effects of GBE on the cardiac histomorphological changes in diabetic ApoE^−/−^ mice. (a) H&E staining of cross-sectional tissue slices of the myocardium. (b) Masson's staining of collagen in the myocardium. The blue area against the red represents collagen deposition. Arrows indicate interstitial fibers. (c, d) Immunostaining of cleaved caspase-3. The brown-yellow area represents the positive expression of cleaved caspase-3. Images of the samples incubated only with the secondary antibody were provided correspondingly. (1A, 1B) Control group; (2A, 2B) untreated diabetic group; (3A, 3B) atorvastatin group; (4A, 4B) 200 mg/kg/day GBE group; and (5A, 5B) 400 mg/kg/day GBE group. (A) 100x magnification; (B) 200x magnification. ^∗^*P* < 0.05 and ^∗∗^*P* < 0.01 versus the untreated diabetic group.

Similar to the findings reported by Ahmed et al. [19], H&E staining showed diffuse disruption of the myocardium, with a fragmented and feathery appearance of DCM. Fibroblasts and inflammatory cells infiltrated the untreated diabetic myocardium, whereas atorvastatin and GBE treatment alleviated their infiltration (Figure 5(a)). In addition, Masson's staining showed that GBE treatment blunted the total cardiac collagen content (Figure 5(b)).

Immunostaining showed that cleaved caspase-3 expression significantly increased in the untreated diabetic mice, compared to that in the control group, whereas atorvastatin and GBE at 200 and 400 mg/kg/day significantly decreased the expression of cleaved caspase-3 (*P* < 0.05, Figures 5(c) and 5(d)). The difference between low-dose and high-dose GBE was not statistically significant.

### 3.5. Effect of GBE on mRNA Levels of Collagen I and III

**Figure 6:**
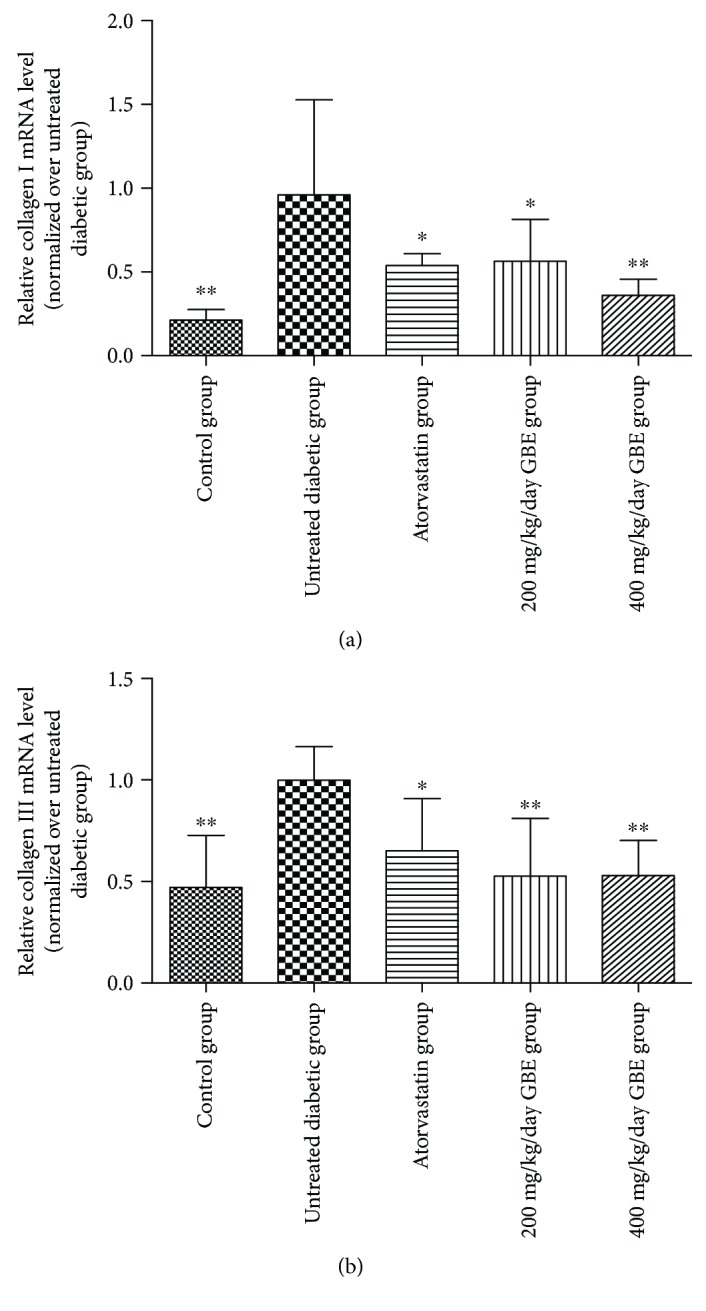
Relative mRNA expression of collagen I and III (normalized over the untreated diabetic group). ^∗^*P* < 0.05 and ^∗∗^*P* < 0.01 versus the untreated diabetic group.

Collagen I and III mRNA levels increased in the untreated diabetic mice. Atorvastatin and GBE (200 and 400 mg/kg/day) treatment resulted in a statistically significant decrease in collagen I and III mRNA levels (*P* < 0.05, Figures 6(a) and 6(b)). There was no significant difference between low-dose and high-dose GBE.

### 3.6. Effect of GBE on Intramyocardial Inflammation

**Figure 7:**
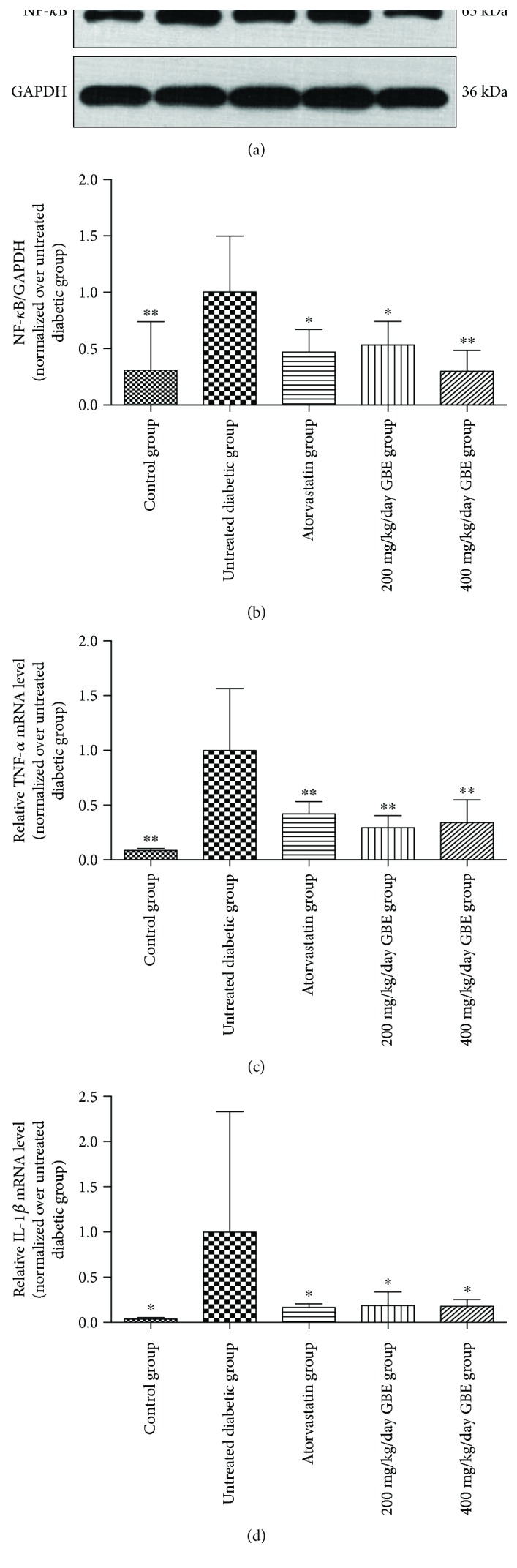
Effects of GBE on NF-*κ*B-mediated intramyocardial inflammation. (a, b) NF-*κ*B expression was determined by Western blotting. (c, d) Relative mRNA expression of TNF-*α* and IL-1*β* measured by real-time PCR. ^∗^*P* < 0.05 and ^∗∗^*P* < 0.01 versus the untreated diabetic group.

NF-*κ*B plays a crucial role in the regulation of intramyocardial inflammation in the development of DCM. The untreated diabetic mice displayed increased expression of NF-*κ*B. Atorvastatin and GBE significantly decreased the expression of NF-*κ*B (*P* < 0.05, Figures 7(a) and 7(b)). TNF-*α* and IL-1*β* mRNA levels increased in the untreated diabetic mice; however, GBE treatment at doses of 200 and 400 mg/kg/day significantly inhibited the STZ-induced increase in TNF-*α* and IL-1*β* mRNA levels (*P* < 0.05, Figures 7(c) and 7(d)). The difference between the findings for low-dose and high-dose GBE was not statistically significant.

### 3.7. Effect of GBE on Hallmarks of ERS-Associated Apoptosis

**Figure 8:**
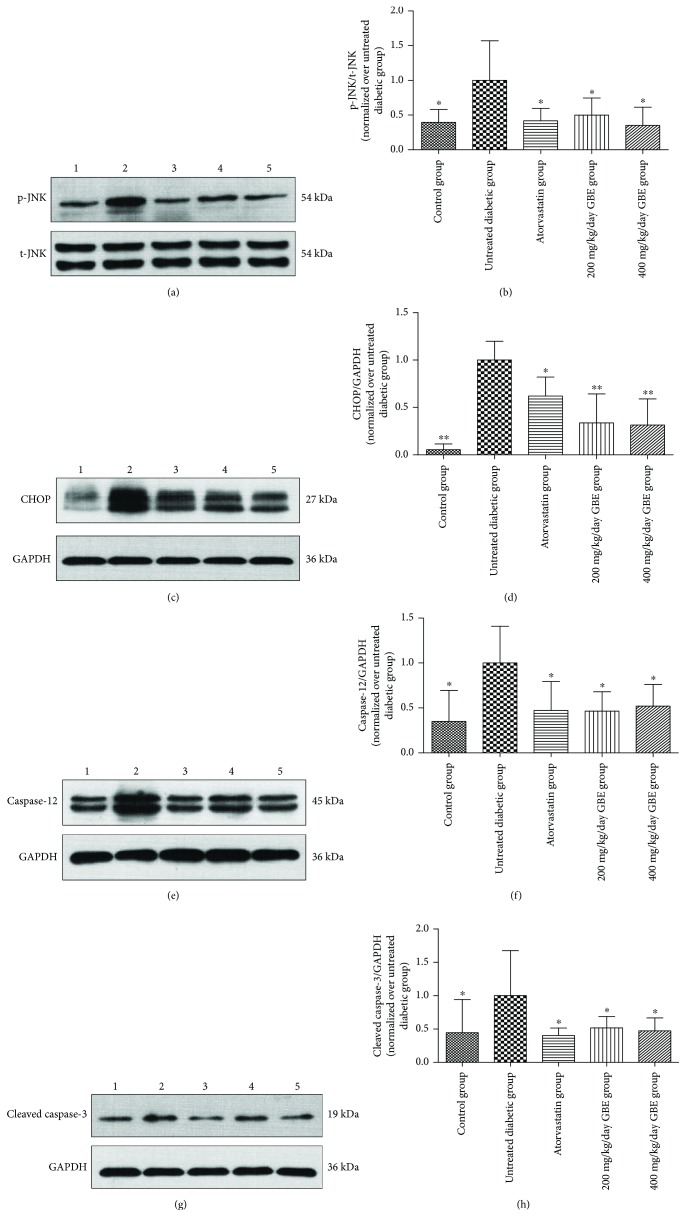
Effects of GBE on ERS-related apoptosis hallmark expression. Expression levels of p-JNK/JNK, CHOP, caspase-12, and cleaved caspase-3 were determined by Western blotting. The expression levels of CHOP, caspase-12, and cleaved caspase-3, were adjusted for GAPDH, and the expression of p-JNK was adjusted for total JNK. These values were normalized over the untreated diabetic group. (a, b) Expression levels of p-JNK/JNK; (c, d) expression of CHOP; (e, f) expression of caspase-12; and (g, h) expression of cleaved caspase-3. ^∗^*P* < 0.05 and ^∗∗^*P* < 0.01 versus the untreated diabetic group. 1: control group; 2: untreated group; 3: atorvastatin group; 4: 200 mg/kg/day GBE group; and 5: 400 mg/kg/day GBE group.

Western blot analysis showed that the expression of the hallmarks of ERS-associated apoptosis, including p-JNK, CHOP, caspase-12, and cleaved caspase-3, significantly increased in the myocardium of diabetic mice, compared to those in the normal control group. This suggested that the p-JNK, CHOP, and caspase-12 cascades were activated in the diabetic myocardium. Atorvastatin and GBE (200 and 400 mg/kg/day) significantly decreased the expression of p-JNK, CHOP, caspase-12, and cleaved caspase-3 (*P* < 0.05, Figures 8(a)–8(h)). There were no statistical differences between the low-dose and high-dose GBE.

## 4. Discussion

Diabetes mellitus is a worldwide metabolic disease responsible for increased morbidity and mortality. Patients with diabetes mellitus are at a high risk of cardiovascular diseases, such as atherosclerosis and DCM, which are comorbidities of diabetes mellitus [20]. STZ has been frequently used to induce diabetes in experimental animals because of its toxic effects on the pancreatic *β*-cells and its potential to induce oxidative stress [21]. Hence, in the present study, we established a diabetic myocardial injury ApoE^−/−^ mouse model by STZ injection combined with a high-fat diet, as previously described [22, 23]. Although it was not able to distinguish between hyperlipidemia- and diabetes-induced myocardial injury, this study aimed to investigate whether GBE attenuated diabetic myocardial injury in ApoE^−/−^ mice, thus providing potential evidence for the treatment of diabetes patients with DCM and atherosclerosis as comorbidities. We found that collagen I and III mRNA expression was elevated in diabetic mice. TNF-*α* and IL-1*β* mRNA levels, which represent intramyocardial inflammation, increased in a diabetic heart owing to increased NF-*κ*B activation. Additionally, hallmarks of ERS-related apoptosis, including p-JNK, CHOP, caspase-12, and cleaved caspase-3, were upregulated in the diabetic heart. These indicated that interstitial collagen deposition, ERS-related apoptosis, and NF-*κ*B-mediated inflammation were induced in diabetic ApoE^−/−^ mice. Consistent with the results of previous studies [15, 16, 19, 24], atorvastatin treatment improved the histological abnormalities, fibrosis, and apoptosis of cardiomyocytes via inhibition of NF-*κ*B-induced inflammation and cleaved caspase-3-mediated apoptosis in the diabetic heart. To our knowledge, the present study is the first study to show that traditional Chinese medicine, GBE, could protect against diabetic myocardial injury, particularly apoptosis, myocardial fibrosis, and NF-*κ*B-mediated inflammation via inhibition of ERS-related apoptosis, as evidenced by the decrease in p-JNK, caspase-12, and cleaved caspase-3 expression. Furthermore, GBE regulated the lipid profile and blood glucose levels.

Cell death, including necrosis and apoptosis, in response to hyperglycemia has been defined as one of the important pathophysiological features of diabetic myocardial injury [1]. Cardiomyocyte loss, myocardial fibrosis, and inflammation result in myocardial remolding that leads to compromised cardiac function. In line with the results of previous studies, the present study showed that hyperglycemia triggered apoptosis of cardiomyocytes. The UPR is an adaptive process to restore the normal ER function. However, the excess and prolonged UPR in hyperglycemic conditions induces cardiomyocyte apoptosis. Three proapoptotic pathways are associated with ERS [4]. The first apoptotic pathway involves activation of JNK by the IRE-1-tumor necrosis factor receptor-associated factor 2- (TRAF2-) apoptosis signal-regulating kinase-1 (ASK1) complex. The second apoptotic pathway is the caspase-12 pathway in rodents. Clustering of caspase-12 in the endoplasmic reticulum membranes may be attributable to TRAF2 recruitment by activated IRE-1 and PERK [25]. Activated caspase-12 translocates from the ER to the cytosol, where it cleaves procaspase 9, and subsequently activates the downstream effector caspase-3. CHOP, which is regulated by IRE1, ATF6, and particularly PERK, is the third proapoptotic pathway related to ERS. Caspase-12 and CHOP are considered specific apoptotic pathways associated with ERS [26]. The present study showed that the three proapoptotic pathways related to ERS were upregulated in the untreated diabetic ApoE^−/−^ mice, accompanied by increased expression of cleaved caspase-3, an effector of caspase-12. Our findings are consistent with the results of Zhang et al. [4], who showed that both the mRNA and protein levels of caspase-12 and CHOP were upregulated in STZ-induced diabetic rats. Taken together, ERS plays an important role in diabetic myocardial injury [27].

An earlier study conducted by Fitzl et al. [28] showed that EGb761, a standard GBE, attenuated the decrease in the volume fraction of myofibrils. According to Qiao et al. [17], GBE could attenuate ischemia/reperfusion-induced cardiac myocyte apoptosis by inhibiting cytochrome c release from the mitochondria and blocking the activation of caspase-3. The present study revealed that GBE downregulated the expression of p-JNK, CHOP, caspase-12, and cleaved caspase-3, indicating that GBE exerted antiapoptotic effects by attenuating ERS.

**Figure 9:**
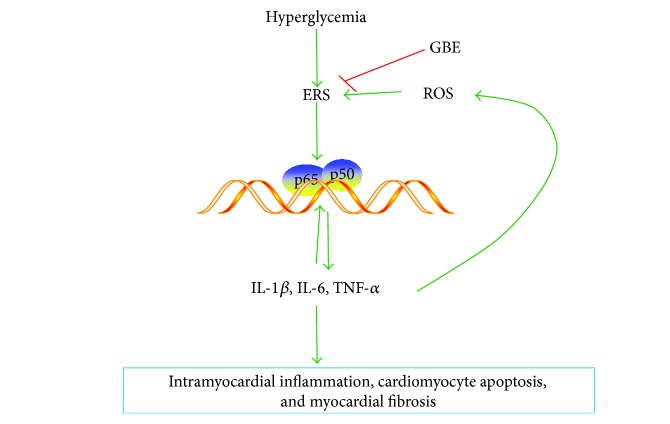
Mechanism underlying the protective effects of GBE against myocardial injury induced by hyperglycemia. The three pathways of UPR could activate NF-*κ*B by phosphorylation of IKK, promoting the production of inflammation cytokines. TNF-*α* activates NF-*κ*B in the presence of ROS in a positive feedback loop, resulting in the generation of more inflammatory cytokines, leading to intramyocardial inflammation, cardiomyocyte apoptosis, and myocardial fibrosis. GBE attenuated diabetic myocardial injury via blocking ERS. ROS: reactive oxygen species.

It has been reported that the IRE1*α* and PERK pathways of the UPR could trigger the NF-*κ*B-mediated inflammatory pathway by phosphorylation of IKK; in addition, the ATF6 pathway is linked to NF-*κ*B activation, suggesting that the three pathways of the UPR could activate this inflammatory cascade [5, 6, 29]. Nuclear translocation of NF-*κ*B results in increased expression of the downstream proinflammatory cytokines, including IL-6, IL-1*β*, and TNF-*α*. IL-1*β* and TNF-*α* further activate ERS (a positive loop), resulting in a more potent inflammatory response, myocardial interstitial fibrosis, and cardiomyocyte apoptosis. TNF-*α* promotes reactive oxygen species (ROS) production and inflammation [30] and mediates JNK activation leading to caspase-3 activation and cardiac cell apoptosis [14]. Therefore, inflammation can also induce ERS [31] (Figure 9). In the present study, GBE decreased collagen type I and III mRNA levels and interstitial collagen deposition, as revealed by Masson's staining. The increase in TNF-*α* and IL-1*β* mRNA levels in the diabetic heart was inhibited by GBE at 200 and 400 mg/kg/day. This was associated with reduced NF-*κ*B expression. Collectively, our findings suggest that GBE attenuated interstitial fibrosis via inhibition of inflammation and ERS, as well as owing to its direct radical scavenging activity and ability to inhibit the opening of the mPTP channel [32]. Although it was not possible to establish whether GBE directly attenuated ERS or acted via modulation of inflammation, it was definite that GBE blocked the positive loop of ERS and inflammation via attenuation of ERS (Figure 9).

In the present study, we observed that GBE at 200 and 400 mg/kg/day decreased plasma glucose levels at the end of the study period. GBE at 200 mg/kg/day decreased the serum glucose level before the mice were sacrificed; additionally, GBE at 400 mg/kg/day could decrease the serum glucose levels; however, it did not reach statistical significance. Cheng et al. [9] showed that GBE restored the activities of antioxidant enzymes, including superoxide dismutase (SOD), catalase (CAT), and glutathione peroxidase (GSH-Px) in the liver and pancreas of STZ-induced diabetic rats. Other studies showed that GBE could alleviate STZ-induced pancreatic damage in mice via inhibition of pancreatic inflammation and expression of proinflammatory cytokines, such as IL-1*β*, TNF-*α*, and IL-6 [10, 33]. The decrease in the serum levels of IL-6, IL-1*β*, and TNF-*α* by GBE treatment in our study suggested that the hypoglycemic effects were partly attributable to the inhibition of inflammation.

In the present study, STZ-induced diabetic ApoE^−/−^ mice displayed severe hyperlipidemia, characterized by elevated serum LDL-c, TC, and TG levels, which were reversed by GBE administration, suggesting that GBE could lower lipid levels in diabetic settings. Zhang et al. [34] reported that GBE exhibited multidirectional lipid-lowering effects in rats, including reduction of cholesterol absorption, inactivation of 3-hydroxy-3-methylglutaryl-coenzyme A (HMG-COA), and favorable regulation of the profiles of essential polyunsaturated fatty acids. Yao et al. [35] showed that GBE at doses of 48 and 96 mg/kg/day normalized ethanol-induced dysregulation of the lipid profiles in rats. According to the study conducted by Cheng et al. [9], the lipid-lowering effects were probably attributed to the improvement of insulin resistance.

In conclusion, GBE attenuated diabetic myocardial injuries, including cardiomyocyte apoptosis, interstitial fibrosis, and intramyocardial inflammation in ApoE^−/−^ diabetic mice by inhibiting ERS. Interestingly, in the present study, the cardioprotective effects of GBE in diabetic conditions were not dose-dependent, which might be because at doses of 200–400 mg/kg/day, the anti-inflammatory and antiapoptotic efficacy of GBE reached a plateau.
